# Current and Future Developments in Imaging and Treatment of White Matter Disease: A Systematic Review

**DOI:** 10.7759/cureus.51030

**Published:** 2023-12-24

**Authors:** Sagar N Malani, Sourya Acharya, Samarth Shukla

**Affiliations:** 1 Department of Medicine, Jawaharlal Nehru Medical College, Datta Meghe Institute of Higher Education and Research (Deemed to Be University), Wardha, IND

**Keywords:** cognitive impairment, white matter lesions, dementia, white matter disease, leukoaraiosis

## Abstract

The elderly often suffer from "mild" dementia due to white matter disease, which is another name for repeated brain infarctions. The degeneration of white matter, which links various parts of the brain to the spinal cord, is the root cause of this disorder, which develops with age. Dementia, imbalance, and movement problems are symptoms of this degenerative disease that worsen with age. This research’s goal is to study current therapy options and identify methods for early diagnosis of white matter illness. The Preferred Reporting Items for Systematic Reviews and Meta-Analyses statement for meta-analyses and systematic reviews served as the basis for our literature review. Results from the search in ScienceDirect and Medline/Pubmed led to the finalization of 33 studies. The complex relationship between white matter hyperintensities (WMHs) and neurological disorders is the subject of this comprehensive review, which sheds light on the varied terrain of WMH studies by highlighting their consequences and developing evaluation techniques.

## Introduction and background

Many white matter hyperintensities (WMHs), often called leukoaraiosis, white matter lesions, or white matter illness, are found during magnetic resonance imaging (MRI) scans. Hyperintense regions are visible on several imaging sequences, such as proton density-weighted, fluid-attenuated inversion recovery (FLAIR), and T2-weighted scans. Those at risk for cardiovascular disease and experiencing signs of cerebrovascular disease often have these WMHs. These hyperintensities are linked to a decrease in functional capacity, dementia, and ultimately death. But they are also popping up more frequently in the brain scans of elderly patients who are otherwise healthy and getting routine clinical testing. This is because, for many age-related neurological disorders, an MRI of the brain serves as the diagnostic tool of choice. To gain a better grasp of their diagnostic as well as prognostic significance in both sick and healthy populations, it is very necessary to enhance the evaluation of WMH [1]. This review gives a thorough summary of recent progress in imaging and treatment for white matter disorders, laying the groundwork for understanding how diagnosis and treatment tactics are evolving.

## Review

Methodology

Inclusion Criteria

Research on ''white matter lesions'' and ''white matter hyperintensities'' as well as other forms of white matter disease conducted between the years 2000 and 2023 will be included in this comprehensive study. To make sure it's relevant, the review includes papers that focus on treatments and procedures related to white matter illness.

Exclusion Criteria

To begin, we omitted studies that were not published in English to ensure linguistic consistency. For the sake of relevance and focus, we also excluded research that did not meet the review's stated goals. Also not included were items that could only be found in books, abstracts, or conference proceedings.

Search Strategy

A variety of search strings were created by combining various keywords. There were a lot of references to white matter disease in Medline/PubMed to locate these studies.

Study Selection

There was a two-pronged approach to choosing the studies. At first, relevant publications were located by employing keywords. We gathered important information after reviewing titles and abstracts to make sure they were relevant and eligible. The second step comprised the independent evaluation of full-text articles. Two reviewers independently checked the publications, and full texts were retrieved from PubMed. Before studies were included in this evaluation, any discrepancies were discussed and agreed upon.

Quality Assessment

Articles that met the inclusion criteria were subjected to a thorough evaluation by two reviewers. Extensive analysis was conducted on the quality and source databases of each article. The PubMed index included those papers. To guarantee that quantitative and qualitative studies were thoroughly considered, The Mixed Methods Assessment Tool (MMAT) evaluated the quality of the publications included in the systematic literature review [2]. A summary of the several studies is provided in the MMAT table found in Table 1.

**Table 1:** Highlighting MMAT's summary MMAT: Mixed Methods Assessment Tool

Category of study designs	Methodological quality criteria	Responses			
		Yes	No	Can’t tell	Comments
Screening questions (for all types)	S1. Are there clear research questions?	✔			
	S2. Does the collected data allow me to address the research questions?	✔			
	Further appraisal may not be feasible or appropriate when the answer is "No" or "Can’t tell" to one or both screening questions.				
1. Qualitative	1.1. Is the qualitative approach appropriate to answer the research question?	✔			
	1.2. Are the qualitative data collection methods adequate to address the research question?	✔			
	1.3. Are the findings adequately derived from the data?	✔			
	1.4. Is the interpretation of results sufficiently substantiated by data?	✔			
	1.5. Is there coherence between qualitative data sources, collection, analysis, and interpretation?	✔			
2. Quantitative randomized controlled trials	2.1. Is randomization appropriately performed?	✔			
	2.2. Are the groups comparable at baseline?	✔			
	2.3. Are there complete outcome data?	✔			
	2.4. Are outcome assessors blinded to the intervention provided?	✔			
	2.5 Did the participants adhere to the assigned intervention?	✔			
3. Quantitative non-randomized	3.1. Are the participants representative of the target population?	✔			
	3.2. Are measurements appropriate regarding both the outcome and intervention (or exposure)?	✔			
	3.3. Are there complete outcome data?	✔			
	3.4. Are the confounders accounted for in the design and analysis?				
	3.5. During the study period, is the intervention administered (or exposure occurred) as intended?	✔			
4. Quantitative descriptive	4.1. Is the sampling strategy relevant to address the research question?	✔			
	4.2. Is the sample representative of the target population?	✔			
	4.3. Are the measurements appropriate?	✔			
	4.4. Is the risk of nonresponse bias low?	✔			
	4.5. Is statistical analysis appropriate to answer the research question?	✔			
5. Mixed methods	5.1. Is there an adequate rationale for using a mixed methods design to address the research question?				
	5.2. Are the different components of the study effectively integrated to answer the research question?				
	5.3. Are the outputs of the integration of qualitative and quantitative components adequately interpreted?				
	5.4. Are divergences and inconsistencies between quantitative and qualitative results adequately addressed?				
	5.5. Do the different components of the study adhere to the quality criteria of each tradition of the methods involved?				

Data Extraction

The two reviewers collaborated to reach a consensus after independently extracting data from each study. White matter disease, detection methods, and comparison studies of different methodologies were covered in the extracted data. The results of the studies were used to reach conclusions in this review. The extraction process was based on primary sources, and any cited references were checked for cross-referencing purposes. It is worth mentioning that the chosen study already contained a summary of these references.

Finalization of references and study characteristics

PRISMA Sheet and the Summary of Final Studies Included in the Review

**Figure 1:**
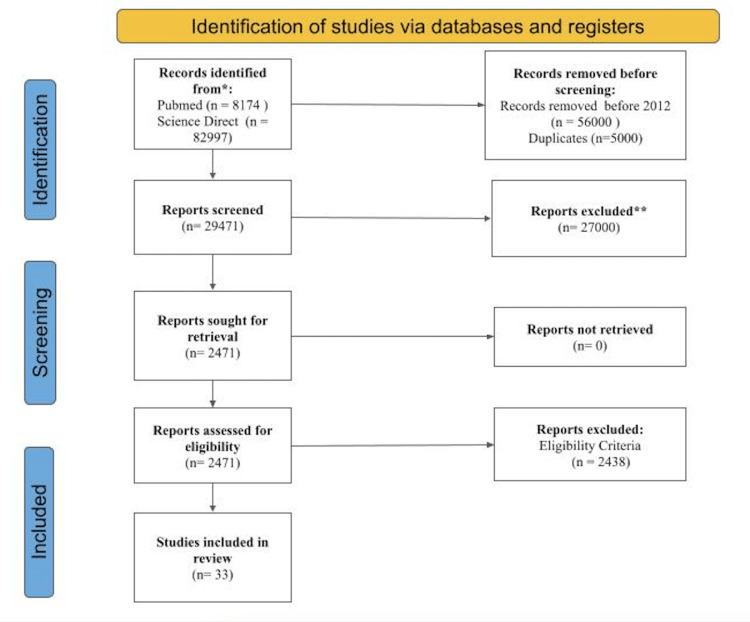
PRISMA diagram PRISMA: Preferred Reporting Items for Systematic Reviews and Meta-Analyses

Both databases were searched using a variety of custom-designed search strings. We found a total of 82,297 references in ScienceDirect and 8174 in Pubmed. A large number of duplicate and non-specific references were returned by the search strings in ScienceDirect. After the first stage, we checked the references for duplication and determined when the study was conducted. Based on these findings, a total of 61,000 references were removed. All things considered, 29471 references were considered for further action. Language, accessibility, book chapters, abstracts, and conference proceedings were among the criteria used to weed out around 27,000 of the 29471 references. For the study, only 2471 were taken into account. The final list of 33 references for the systematic literature review was determined by considering the study's relevance to the task and its objectives. A summary of the PRISMA document is given in Figure 1.

Study Characteristic Table

 A summary of the included references' characteristics is given in Table 2.

**Table 2:** Highlighting the study characteristics of included references BIANCA: Brain Intensity AbNormality Classification Algorithm; CBI: Covert brain infarction; WMD: White matter dementia; PD: Parkinson's disease; MS: Multiple sclerosis; WML: White matter lesion; CL: Cortical lesions; DTI: Diffusion tensor imaging; AVD: Atherosclerotic vascular disease; WMH: White matter hyperintensity; CMB: cerebral microbleeds; ICH: Intracerebral hemorrhage; OASIS-AD: Automated Statistical Inference for Segmentation-Alzheimer's disease; PML: Progressive multifocal leukoencephalopathy; PML-IRIS: Progressive multifocal leukoencephalopathy-immune reconstitution inflammatory syndrome; MRI: Magnetic resonance imaging; PET: Positron emission tomography; QSI: Q-space diffusional MRI; NAWM: Normal-appearing white matter; DAWM: Diseased-appearing white matter; FA: Fractional anisotrop; PKU: Phenylketonuria; QSM: Quantitative susceptibility mapping; FLAIR: Fluid-attenuated inversion-recovery; WM: White matter; rt-PA: Recombinant tissue plasminogen activator; BCS: Baló's concentric sclerosis

Reference	Nature of study	Total number of patients	Detection technique/therapy	Characteristics
Griffanti et al., 2016 [1]	Quantitative study	20	Detection of white matter hyperintensities	Validated and optimized BIANCA on two datasets using distinct patient populations and MRI protocols
Morovic et al., 2019 [3]	Review article	None	Early disease detection of dementia	Control of vascular risk factors
Leung et al., 2021 [4]	Population‐based study	2, 41, 050	Identifying dementia related to id-CBI and id-WMD	On the neuroimage report, white matter sickness was categorized as mild, moderate, severe, or indeterminate; natural language processing was also used to identify WMD and accidentally discovered covert brain infarction (id-CBI)
Wu et al., 2023 [5]	Population‐based study	50	Detection of regions of greater WMH in the PD	Comparison of WMH volume of each region with the corresponding region in the control group
Ong et al., 2022 [6]	Comparative study	32	Recognizing morphology unique to genuine WML and detecting textural characteristics	Introduced a technique for the automated segmentation of light WML loads utilizing a random forest (RF) classifier, an intensity standardization methodology, and an integrated clustering technique called grey level co-occurrence matrix (GLCM)
Tillema et al., 2017 [7]	Evidence-based study	28	Detection of connectivity between cortical and white matter lesions in early MS	CL and WML maps were created, and DTI was used to calculate inter-lesional connectivity and volumetric connectivity indices
Bijanki et al., 2013 [8]	Imaging study	57	Identification of variations in fractional anisotropy (FA) between the healthy and AVD comparison (HC)	Determine fractional anisotropy-prone areas for white matter injury (FA)
Zhang et al., 2022 [9]	Cross-sectional study	144	Detection of regional cerebral blood flow	A circadian blood pressure monitor, which measures the blood pressure rhythm, and imaging of the brain's perfusion
Rane et al., 2020 [10]	Population‐based study	152	Detection of the burden and spatial distribution (periventricular vs. deep) of WMH	Novel MRI techniques for multiple axis and cognitive connections
Capuana et al., 2021 [11]	Retrospective study	434	Determining the relationship between rt-PA, CMBs, white matter damage, and ICH during intravenous thrombolysis (IVT).	We used multivariate regression analysis to find out how CMB and WMD affected different ICH subgroups
Ding et al., 2020 [12]	Comparative study	20	Detection of automatic lesion segmentation in multiple sclerosis	OASIS-AD is proposed, with rigorous feature space creation and logistic regression as the basis
Igra et al., 2017 [13]	Review article	None	Diagnosis of multiple sclerosis (MS) and elucidate the imaging features of psychiatric symptoms (e.g., PML and PML-IRIS)	Demonstrating McDonald's Criteria in order to aid the diagnosis of MS
Moghekar et al., 2012 [14]	Prospective study	50	Finding a connection between MRI-detected cerebral white matter degeneration and autopsy-confirmed Alzheimer's illness	Blinded assessment of white matter disorders in magnetic resonance imaging (MRI) images taken before and after death according to the standards established by the Cardiovascular Health Study (CHS)
Pietroboni et al., 2022 [15]	Exploratory study	33	Detection of amy-PET uptake in white matter lesions (WML) and in white matter that appears normal (NAWM) in individuals with Alzheimer's disease (AD) and dementias that are not associated with AD.	People with cognitive impairments were tested using MRI scans of the brain, Amy-PET scans of the cerebrospinal fluid, and Aβ1-42 (Aβ) findings
Fujiyoshi et al., 2016 [16]	Clinical study	Animal-based	Detection of myelin signals	Enhance QSI methods so they may be used in healthcare situations
West et al., 2014 [17]	Prospective study	35	NAWM and DAWM identification in MS patients	We used a 1.5 T Philips MR-scanner for our qMRI and qMRS studies
Wang et al., 2012 [18]	Cross-sectional study	92	Detection of FA and brain volume	Whole-brain DTI, T1-weighted structural MRI, and neuropsychological assessment
Dadar et al., 2017 [19]	Comparative study	201	Detection of WMHs from MRI data	10 different linear and nonlinear classification methods evaluated
Hellewell et al., 2021 [20]	Cohort study	63	Detection of abnormalities in PKU	Adults diagnosed with PKU were studied using diffusion kurtosis and diffusion MRI metrics
Vanderbecq et al., 2020 [21]	Comparative study	147	Detection of white matter hyperintensities (WMHs)	Evaluation of the dice similarity coefficient (DSC) segmentation accuracy comparing the automated and manual software
Kan et al., 2022 [22]	Exploratory study	24	Finding microstructural white matter alterations in Parkinson's disease patients associated with cognitive decline (PD)	R2* relaxometry analysis in conjunction with QSM
Waymont et al., 2020 [23]	Comparative study	243	Detection of brain white matter hyperintensities of presumed vascular origin	To find the best algorithm, we tested two automatic methods with visual and manual segmentation, and we used the open-source lesion segmentation toolbox (LST)
Tran et al., 2022 [24]	Comparative study	60	Detection of MS lesions and age-related WMH	Introducing the modified White matter Hyperintensities Automatic Segmentation Algorithm for 3D T2-FLAIR datasets (WHASA-3D)
Balakrishnan et al., 2021 [25]	Systematic review	None	Detection of white matter hyperintensities	A systematic review of fully automatic WMH quantification methods
Rutherford and Hamilton, 2019 [26]	Review article	Animal-based	Therapy for most common leukodystrophies	Critical animal models and their role in the discovery of new therapeutic approaches
Abbink et al., 2019 [27]	Clinical study	Animal-based	Therapy for Vanishing white matter (VWM)	Modulation of eIF2B activity in ameliorating an altered translation and improving the disease
Tzanetakos et al., 2020 [28]	Retrospective study	8	Therapy for Baló’s concentric sclerosis (BCS)	By stressing MRI traits and therapeutic maneuver differences, we propose a new potential BCS categorization and provide therapeutic concepts that are based on the characteristics of each BCS subgroup
Rosenberg et al., 2021 [29]	Dose-escalating safety/toxicology study	8	Gene therapy for metachromatic leukodystrophy	Risk identification of 12 locations in the white matter of non-human primates' central nervous systems for intraparenchymal administration of the AAVrh.10hARSA vector
Dixit et al., 2017 [30]	Review article	None	Therapy for multiple sclerosis	Efficacy of helminth treatment in animal experiments and preliminary and ongoing human medical trials
Bradbury et al., 2021 [31]	Review article	None	Therapy for Krabbe disease	A synopsis of Krabbe disease's past, present, and future, including the changing landscape of treatment options (both alone and in combination), the discovery of novel pathogenic pathways, and the outcomes of these endeavors
Sriwastava et al., 2021 [32]	Review article	194 patients from 62 articles	Therapy for MS-related PML	Discover what variables people with MS-related PML have in common to have a good prognosis
Eichler et al., 2017 [33]	Phase 2-3 safety and efficacy study	17	Hematopoietic stem-cell gene therapy for cerebral adrenoleukodystrophy	IV injection of autologous CD34+ cells transduced with elivaldogene tavalentivec (Lenti-D) lentiviral vector to track MRI lesion magnitude, neurological function alterations, and graft-versus-host disease, death, and major functional impairments
Stefaniak et al., 2018 [34]	Cohort study	149	Therapy for Fabry disease (FD)	In order to investigate the relationship between Fabry disease (FD) patients' clinical features, the rate of development of WMH, and enzyme replacement treatment (ERT)

Results

Methods for Detection of White Matter Disease

Multiple methods have been devised to diagnose white matter disorders. Using the k-nearest neighbor (k-NN) algorithm, a supervised completely automated system called the Brain Intensity Abnormality Classification Algorithm (BIANCA) can detect WMH. Integrating BIANCA into FSL provides a dependable and automated technique for WMH segmentation, which is especially helpful in large cohort investigations [1]. Prevention of cognitive decline must be prioritized due to the impending tripling of dementia cases by 2050. Using ultrasonography or biomarkers, cutting-edge studies are attempting to intervene twenty years before clinical signs appear, reshaping the conventional wisdom that Alzheimer's disease (AD) is only a problem for the elderly [3]. Dementia is associated with 29.0% incidentally discovered id-white matter dementia (WMD) and 4.7% id-covert brain infarction (CBI), according to population-based magnetic resonance imaging (MRI) screening [4]. The younger generation is particularly vulnerable to this danger. Histological analysis reveals a variety of pathological alterations when WMHs on T2-FLAIR MRI indicate small vascular disease [5]. Greater occipital WMH is associated with worsening motor symptoms in Parkinson's disease (PD), but bigger, longer-term trials are needed to fully evaluate their long-term effects.

Manual, subjective measures and variability make quantifying WMLs hard. Computer-assisted techniques provide automatic, repeatable measurement. A novel method that automates mild WML segmentation with intensity standardization is based on the similarity between WMLs and ischemia on FLAIR MRI, which is seen in MS. The use of RF classifiers with grey-level co-occurrence matrix-embedded clustering for texture and morphology recognition was the subject of one study [6]. An additional study uses provincial MRI to try to clarify the hazy connection between multiple sclerosis (MS) white matter lesions (WMLs) and cortical lesions (CLs). Although there seems to be a link in pathology, how exactly CLs and WMLs contribute to the start and development of diseases is still up for debate. The research finds that there is direct communication between WMLs and CLs as well as among WMLs themselves. More study is needed to understand the connections between lesions and the brain in MS, particularly in the treatment-resistant progressive stage [7].

Atherosclerotic vascular disease (AVD) impacts brain health, particularly white matter (WM), in developed areas. Nevertheless, diffusion tensor imaging (DTI) has not been utilized to study the effects of AVD on the white matter at this time. Examining the variations in FA between AVD patients and a healthy control group was the primary goal of this study. The results showed that white matter integrity varied among AVD older persons. Significant group effects were observed in all examined brain locations except the temporal lobes; moreover, older people had reduced FA in numerous regions [8]. Blood pressure rhythm (BPR) and blood pressure variability (BPV) increase the risk of WMLs, which have recently been linked to hypertension and aging. Reducing regional cerebral blood flow was associated with increased BPV and rhythm abnormalities in individuals with WMLs [9], indicating that disruptions in BPR or increased BPV may impede WML advancement through this mechanism.

The presence of WMHs revealed by MRI is a typical way to diagnose cerebrovascular illness, which is more prevalent in the elderly. Although there is evidence that WMHs can alleviate cognitive symptoms, it is unclear how exactly they relate to cognitive performance or other degenerative diseases. Significant relationships with Montreal Cognitive Assessment, Trail making test, and category fluency test performance were found in focused research on WMH extent and spatial distribution (periventricular vs. deep) [10]. WMD, intracerebral hemorrhage (ICH) following intravenous thrombolysis, and recombinant tissue plasminogen activato were the subjects of another study. Based on the research, the existence of CMBs and their location in the deep or infratentorial zones were predicted to cause large hemorrhagic changes. Nevertheless, these variables did not indicate the likelihood of ICH symptoms or negative results [11].

Though structural magnetic resonance imaging plays a very important role in the diagnosis of neurodegenerative disorders like AD in the elderly, the application of automated MRI processing to their images is hindered by age-related pathologies such as WMHs and atrophy. The older adult automated WMH segmentation approach Automated Statistical Inference for Segmentation-Alzheimer's disease (OASIS-AD) fared better than the other methods, including Method for Inter-Modal Segmentation Analysis, lesion segmentation tool (LST), and Automated Statistical Inference for Segmentation [12]. The results showed that when it came to WMH, OASIS-AD outperformed the other automatic segmentation methods that are currently available. Since 2001, MRI has been an important tool for the diagnosis of MS, helping to resolve natalizumab-related problems and conform to the new McDonald criteria. Thanks to MRI's changing function, earlier intervention in MS is now possible [13].

The link between cognitive decline in the elderly and white matter diseases found by MRI has prompted concerns regarding causation. White matter scores from the Cardiovascular Health Study (CHS) were significantly correlated with a number of different AD pathology markers, according to research into the pathophysiology of the disease that used MRI and autopsy. Nevertheless, there was no independent correlation between amyloid angiopathy and CHS score, and when AD pathology was taken into account, the correlation between clinical dementia diagnosis and CHS score vanished [14].

Dementia, namely AD, is primarily caused by the accumulation of beta-amyloid (Aβ) in the brain and the aggregation of tau proteins. New research suggests that changes in white matter have a significant role in the development of AD. The use of amyloid tracers in positron emission tomography (PET) allows for the assessment of amyloid deposition in the brain's WM. Using individuals with and without AD dementia, researchers evaluated amy-PET uptake in WML and normal-appearing white matter (NAWM). This shows that amy-PET can identify microstructural white matter damage in non-AD dementia. The potential accumulation of amyloid in this particular context, though, could restrict its utility in evaluating white matter dysfunction in individuals with AD [15].

Recent studies have linked abnormalities in central nervous system white matter to several neurological and behavioral disorders. By measuring non-Gaussian water diffusion, q-space diffusional MRI (QSI) can evaluate biological barriers like myelin sheaths. Nevertheless, the time-consuming nature of QSI limits its clinical relevance. Optimal clinical application of QSI protocols and visualization of myelin signals were the targets of another investigation. A new method, the myelin map, which shows the condition of the central nervous system's myelin, was introduced as a result of the findings. An essential caveat to add is that the human myelin map has not been confirmed histologically [16].

Demyelination is a marker of the inflammatory, chronic, central nervous system (CNS)-affecting MS condition. Finding and tracking lesions, especially with T2-weighted imaging, requires an MRI. A conundrum in clinicopathology exists; however, white matter (WM) abnormalities do not seem to be associated with clinical impairment. Quantitative magnetic resonance imaging (qMRI) and quantitative magnetic resonance spectroscopy (qMRS) were used in a study that compared diseased-appearing white matter (DAWM) and NAWM in patients with typical MS and those with minimal lesions. The results showed pathological changes. We can learn more about the clinicoradiological conflict by examining the correlation between qMRI and clinical status.

A cross-sectional study looked at the effects of amnestic moderate cognitive impairment (MCI) and AD on white matter (WM) [18]. Using whole-brain DTI, T1-weighted MRI, and neuropsychological tests, the study compared older persons with MCI, no psychometric impairment, and healthy controls (HC). Compared to the control group (WM), those with MCI had lower FA and increased radial diffusivity in both parahippocampal WM.

The cognitive complaint group showed a middle-ground pattern, which could mean that DTI can help find neurodegenerative alterations early on. A different study [19] used MRI data to identify WMHs using a variety of classifiers. Even without FLAIR data, random forests consistently outperformed other classifiers across all datasets when it came to identifying WMHs using FLAIR scan data.

The amino acid metabolism can be affected by phenylketonuria (PKU), a genetic disease. The current trend in healthcare is to prioritize short-term phenylalanine level control above long-term evaluation of neuronal damage. Subclinical white matter abnormalities in PKU may be detectable using diffusion kurtosis imaging (DKI), a state-of-the-art MRI technique. When treating an adult PKU cohort, it was common practice to check their phenylalanine levels, do MRIs, and assess their neurocognitive abilities [20]. Severe periventricular white matter alterations were indicated by both siblings' DKI measures, which were higher than controls. Even when no outward symptoms or conventional MRI abnormalities are present, DKI can detect specific, progressive problems with brain diffusion in PKU.

After comparing seven different techniques, nicMSlesion emerged as the clear winner when it came to geriatric WMH segmentation [21]. When considering the dice similarity coefficient (DSC), this became very evident. The study objectively assessed various methods for WMH segmentation. On the Alzheimer's Disease Neuroimaging Initiative study dataset, the deep learning method NicMSlesion outperformed all other approaches with a DSC of 0.595, demonstrating remarkable efficacy. When tested on the clinical routine dataset and data that had artifacts, it fared considerably worse, resulting in an overall performance rating of seventh. When tested on the clinical routine dataset, the top three techniques - BIANCA, lesion prediction algorithm, and multiple sclerosis lesion segmentation - outperformed the competition by a similar margin. The study's results shed light on how various WMH segmentation approaches functioned. Based on these findings, radiologists will be able to choose an appropriate segmentation tool for their datasets. In clinical practice, this makes WMH segmentation more efficient and increases its objectivity.

People with moderate cognitive impairment, HC, and those with normal cognition were all part of the study. To study cognitive impairment in PD, anomalies in white matter were examined using voxels-based R2* relaxometry analysis. According to this study, which looked at how R2* values relate to cognitive function in PD, changes in the R2* values of specific white matter regions might signal the beginning of cognitive decline in PD [22]. Other studies have looked at WMH linked to cerebral small artery disease using the LST and both automated and manual segmentation methods [23]. After comparing the lesion growth algorithm (LGA) to Scheltens' score and human segmentation, it was determined that LGA was the most robust algorithm.

Improving the diagnosis and tracking of MS requires automating the evaluation of WMH load. Using several neurological disorders, like Alzheimer's, frontotemporal dementia, MS, HC, and cognitive deficits, this study aimed to automate WMH segmentation. White matter Hyperintensities Automatic Segmentation Algorithm for 3D T2-FLAIR datasets efficiently and accurately automated age-related WMH and segmented MS lesions without requiring any adjustments [24].

Traditional semi-automatic methods are still favored in clinical research, even if completely automatic WMH quantification methods show promise. A recent complete evaluation of existing methodologies was conducted on WMH presumed to have a vascular origin. There is no clinical evidence to support the advantage of convolutional neural network (CNN) schemes over k-NN algorithm, linear regression, or unsupervised techniques for WMH segmentation [25], despite CNN schemes' broad popularity and accuracy.

Therapies for White Matter Disease

This study’s purpose was to find future treatments for leukodystrophies by reviewing animal models of these disorders [26]. According to the findings, these models have widespread flaws in the existing literature. Among the several leukodystrophies, vanishing white matter (VWM) leukodystrophy is particularly dangerous because it primarily affects youngsters and currently has no treatment or cure. The eukaryotic initiation factor 2B (eIF2B) is a critical regulator of the initiation and regulation of messenger RNA translation during the integrated stress response (ISR), and recessive mutations in this gene cause this disease. Researchers looked into the idea of improving aberrant translation and disease mitigation by changing eIF2B activity [27]. Dysregulation of the ISR was identified as a possible therapeutic target and one of the main pathomechanisms of VWM.

Very uncommon demyelinating disease Baló's concentric sclerosis (BCS) might show up on MRI with a broad variety of symptoms. Separated into three groups, BCS was systematically based on radiological characteristics and treatment outcomes [28]. At the beginning of the disease, six patients showed 2.7 cm in diameter tumefactive BCS lesions, and seven patients had concurrent MS-like plaques on brain MRI. The average age at which the disease started was 26.3 years, and the mean period of follow-up was 56.8 months. Radiological characteristics and treatment response were used to classify patients into three groups: some people with basal cell synovitis fall into one of four categories. Patients with BCS who do not have typical MS lesions are included in group 1. Patients with typical MS lesions are included in group 2. Patients with typical MS lesions are included in group 3. Patients with typical MS lesions are included in group 4. Patients with BCS who react effectively to intravenous methylprednisolone and MS-disease-modifying treatments are included in group 4. At the end of the study, a new BCS category was introduced that takes into account radiological features and treatment results. Although immunosuppressive medications like cyclophosphamide are frequently useful, other treatments including anti-CD20 monoclonal antibodies or conventional disease-modifying MS medications may be considered for BCS with mixed MS-like lesions. To enhance clinical outcomes and refine therapy options, it is essential to validate these results through larger-scale investigations.

Metachromatic leukodystrophy (MLD) is an incredibly rare and disabling neurological condition in children caused by an enzyme defect in arylsulfatase A (ARSA). The research evaluated the safety of MLD treatment in nonhuman primates (NHPs) when delivered directly into their CNS using the AAVrh.10 vector expressing human ARSA (AAVrh.10hARSA) [29]. Administering low-dose AAVrh.10hARSA did not cause any significant negative effects. In contrast, low-dose AAVrh.10ARSA and null vector MRI scans revealed immune cell infiltrates and localized CNS abnormalities at the catheter infusion sites. Another intriguing path for the identification of anti-inflammatory drugs is the investigation of helminth infection as a possible treatment for MS in both animal models and human clinical trials (phases 1 and 2) [30].

One of the most serious metabolic diseases is globoid cell leukodystrophy or Krabbe disease. A review of the literature delves into new pathogenic processes and how they may improve Krabbe disease treatment approaches [31]. This review covers the trend of how Krabbe's illness has evolved throughout time. It examines the evolution of treatments and the potential influence of novel disease processes on future therapeutic choices. Treatments for MS that change the course of the disease are efficient in lowering the frequency as well as the severity of relapses. A meta-analysis and literature review [32] found predictive variables for MS-related progressive multifocal leukoencephalopathy (PML). While there was no clear winner when it came to PML treatments, better results were associated with younger age and a lower John Cunningham virus viral load at the time of diagnosis.

Adrenoleukodystrophy is an X-linked genetic condition that develops when the ABCD1 gene is mutated, resulting in the loss of the ALD protein. Research into possible treatments for neurological impairment and mortality caused by illness development is ongoing. The lentiviral vectors used in these patients' treatments are elivaldogene tavalentivec (Lenti-D) [33]. The results show that Lenti-D gene therapy is a safe as well as effective treatment option for boys with early-stage cerebral adrenoleukodystrophy, similar to allogeneic stem-cell transplantation. Nevertheless, more study is required to find the long-term safety and duration of response. In FD, an X-linked lysosomal storage disorder caused by mutations in the GLA gene leading to α-galactosidase A deficiency, a study looked into how WMH in FD evolved in reaction to ERT and other clinical variables [34]. Although ERT has the potential to reduce the progression of WMH in FD patients, no association between the two was observed in the study.

Discussion

The primary focuses of this research are WMHs, which have recently been the subject of promising imaging and therapeutic developments, and their connections to neurological and neurodegenerative disorders. Innovative methods like ultrasound or biomarker-based vascular evaluations can detect white matter illnesses, especially Alzheimer's, early enough to allow therapies as much as 20 years before clinical symptoms appear, according to the research. The study also highlights the utility of NLP algorithms for facilitating research in this field by investigating the links between WMH, dementia, and cerebrovascular illness. The study also looks at the possibility of gene treatments for white matter diseases, automated segmentation approaches, and several sophisticated imaging methods for assessing WMH. The evidence is consistent with the study's findings. Innovative ways are needed to enable early detection of Alzheimer's and personalized therapies; this highlights the complexity of WMH research and its implications for neurological illnesses. Gene therapy for white matter diseases and the development of new MS treatments are only two of the many potential areas for further study that this study identifies.

To contribute to what is already known, this paper reviews all the recent advances in the study of white matter disorders. Possible diagnostic and treatment approaches can be better understood with its help. Other studies that have looked into the connections between WMHs and various neurological diseases have also found this to be the case. In sum, the findings of this study add to what is already known about white matter illnesses and the consequences they have on patient care and also pave the way for future studies in this area.

The extensive literature review covers a wide range of topics related to WMHs and their links to neurological and neurodegenerative diseases. An innovative study has proposed a clinical method that can detect the start of AD 20 years before symptoms manifest, casting doubt on the long-held belief that the disease only affects the elderly. The importance of detecting vascular abnormalities early by ultrasound or biomarkers cannot be overstated [15].

The purpose of this population-based MRI screening was to investigate covert cerebrovascular disease (CCD), which has been associated with dementia. More common in younger participants, the study indicated that id-WMD and id-CBI were associated with an elevated risk of dementia [10,11]. By using a novel NLP technique, id-CCD may be identified, which streamlines its investigation and improves the risk assessment for dementia in regular neuroimaging reports [4]. Automated WMH segmentation approaches, the link between atherosclerotic vascular disease and white matter integrity, and the influence of WMH on Parkinson's disease are all covered in the review. It discusses QSI for myelin evaluation [16], MRI for MS diagnosis (current state of affairs), and DKI [20] for phenylketonuria white matter abnormality detection. The relevance of WMH research for neurological illnesses and the development of new evaluation tools are illuminated by this thorough analysis.

Leukodystrophies and related illnesses are better understood because of the works reviewed here. In order to create effective treatments, they stress the significance of comprehending the underlying mechanisms [26]. As a potential treatment for disappearing white matter disorder, research into altering eIF2B activity is encouraging [27]. Radiological markers are being used to classify BCSs, which could lead to better treatment personalization. The results of MLD therapy in NHPs are encouraging, but they also indicate how important it is to be careful with large doses [33]. The potential of helminth infection as a therapy for MS is fascinating. Gene therapy is being investigated in the field of adrenoleukodystrophy as a potentially less invasive option [34]. Lastly, ERT's effect on Fabry's disease progression has been the subject of investigation; nevertheless, further study is required in this area.

## Conclusions

The complex relationships between WMH and neurological disorders are examined in this comprehensive review. Ultrasound or biomarker-based vascular evaluations may one day be able to diagnose AD 20 years before any noticeable symptoms appear, according to a novel perspective that questions the current understanding of the disease. The review's findings highlight the importance of incidentally discovered white matter illness and natural language processing algorithms for expedited research, especially in younger individuals, and CCD connections with dementia. A major clinical concern is white matter disease, particularly in vascular dementia, where small vessel disease is a major risk factor. We should look into natural language processing to speed up research, improve automated WMH segmentation, prioritize early Alzheimer's diagnosis, and further examine gene therapy and helminth infection.

## References

[1] Griffanti L, Zamboni G, Khan A (2016). BIANCA (Brain Intensity AbNormality Classification Algorithm): a new tool for automated segmentation of white matter hyperintensities. Neuroimage.

[2] Hong QN, Fàbregues S, Bartlett G (2018). The Mixed Methods Appraisal Tool (MMAT) version 2018 for information professionals and researchers. Educ Inf.

[3] Morovic S, Budincevic H, Govori V, Demarin V (2019). Possibilities of dementia prevention - it is never too early to start. J Med Life.

[4] Leung LY, Fu S, Luetmer PH (2021). Agreement between neuroimages and reports for natural language processing-based detection of silent brain infarcts and white matter disease. BMC Neurol.

[5] Wu H, Hong H, Wu C (2023). Regional white matter hyperintensity volume in Parkinson's disease and associations with the motor signs. Ann Clin Transl Neurol.

[6] Ong K, Young DM, Sulaiman S (2022). Detection of subtle white matter lesions in MRI through texture feature extraction and boundary delineation using an embedded clustering strategy. Sci Rep.

[7] Tillema JM, Weigand SD, Mandrekar J, Shu Y, Lucchinetti CF, Pirko I, Port JD (2017). In vivo detection of connectivity between cortical and white matter lesions in early MS. Mult Scler.

[8] Rowe Bijanki K, Arndt S, Magnotta VA (2013). Characterizing white matter health and organization in atherosclerotic vascular disease: a diffusion tensor imaging study. Psychiatry Res.

[9] Zhang D, He M, He Q, Li Z (2022). Blood pressure rhythm and blood pressure variability as risk factors for white matter lesions: a cross-sectional study. Med Sci Monit.

[10] Rane S, Owen J, Hippe DS, Cholerton B, Zabetian CP, Montine T, Grabowski TJ (2020). White matter lesions in mild cognitive impairment and idiopathic Parkinson’s disease: multimodal advanced MRI and cognitive associations. Journal of Neuroimaging.

[11] Capuana ML, Lorenzano S, Caselli MC, Paciaroni M, Toni D (2021). Hemorrhagic risk after intravenous thrombolysis for ischemic stroke in patients with cerebral microbleeds and white matter disease. Neurol Sci.

[12] Ding T, Cohen AD, O'Connor EE (2020). An improved algorithm of white matter hyperintensity detection in elderly adults. Neuroimage Clin.

[13] Igra MS, Paling D, Wattjes MP, Connolly DJ, Hoggard N (2017). Multiple sclerosis update: use of MRI for early diagnosis, disease monitoring and assessment of treatment related complications. Br J Radiol.

[14] Moghekar A, Kraut M, Elkins W, Troncoso J, Zonderman AB, Resnick SM, O'Brien RJ (2012). Cerebral white matter disease is associated with Alzheimer pathology in a prospective cohort. Alzheimers Dement.

[15] Pietroboni AM, Colombi A, Carandini T (2022). Amyloid PET imaging and dementias: potential applications in detecting and quantifying early white matter damage. Alzheimers Res Ther.

[16] Fujiyoshi K, Hikishima K, Nakahara J (2016). Application of q-space diffusion MRI for the visualization of white matter. J Neurosci.

[17] West J, Aalto A, Tisell A, Leinhard OD, Landtblom AM, Smedby Ö, Lundberg P (2014). Normal appearing and diffusely abnormal white matter in patients with multiple sclerosis assessed with quantitative MR. PLoS One.

[18] Wang Y, West JD, Flashman LA (2012). Selective changes in white matter integrity in MCI and older adults with cognitive complaints. Biochim Biophys Acta.

[19] Dadar M, Maranzano J, Misquitta K (2017). Performance comparison of 10 different classification techniques in segmenting white matter hyperintensities in aging. Neuroimage.

[20] Hellewell SC, Welton T, Eisenhuth K, Tchan MC, Grieve SM (2021). Diffusion kurtosis imaging detects subclinical white matter abnormalities in Phenylketonuria. Neuroimage Clin.

[21] Vanderbecq Q, Xu E, Ströer S, Couvy-Duchesne B, Diaz Melo M, Dormont D, Colliot O (2020). Comparison and validation of seven white matter hyperintensities segmentation software in elderly patients. Neuroimage Clin.

[22] Kan H, Uchida Y, Ueki Y (2022). R2* relaxometry analysis for mapping of white matter alteration in Parkinson's disease with mild cognitive impairment. Neuroimage Clin.

[23] Waymont JM, Petsa C, McNeil CJ, Murray AD, Waiter GD (2020). Validation and comparison of two automated methods for quantifying brain white matter hyperintensities of presumed vascular origin. J Int Med Res.

[24] Tran P, Thoprakarn U, Gourieux E (2022). Automatic segmentation of white matter hyperintensities: validation and comparison with state-of-the-art methods on both multiple sclerosis and elderly subjects. Neuroimage Clin.

[25] Balakrishnan R, Valdés Hernández MD, Farrall AJ (2021). Automatic segmentation of white matter hyperintensities from brain magnetic resonance images in the era of deep learning and big data - a systematic review. Comput Med Imaging Graph.

[26] Rutherford HA, Hamilton N (2019). Animal models of leukodystrophy: a new perspective for the development of therapies. FEBS J.

[27] Abbink TE, Wisse LE, Jaku E (2019). Vanishing white matter: deregulated integrated stress response as therapy target. Ann Clin Transl Neurol.

[28] Tzanetakos D, Vakrakou AG, Tzartos JS (2020). Heterogeneity of Baló's concentric sclerosis: a study of eight cases with different therapeutic concepts. BMC Neurol.

[29] Rosenberg JB, Chen A, De BP (2021). Safety of direct intraparenchymal Aavrh.10-mediated central nervous system gene therapy for metachromatic leukodystrophy. Hum Gene Ther.

[30] Dixit A, Tanaka A, Greer JM, Donnelly S (2017). Novel therapeutics for multiple sclerosis designed by parasitic worms. Int J Mol Sci.

[31] Bradbury AM, Bongarzone ER, Sands MS (2021). Krabbe disease: new hope for an old disease. Neurosci Lett.

[32] Sriwastava S, Kataria S, Srivastava S (2021). Disease-modifying therapies and progressive multifocal leukoencephalopathy in multiple sclerosis: a systematic review and meta-analysis. J Neuroimmunol.

[33] Eichler F, Duncan C, Musolino PL (2017). Hematopoietic stem-cell gene therapy for cerebral adrenoleukodystrophy. N Engl J Med.

[34] Stefaniak JD, Parkes LM, Parry-Jones AR, Potter GM, Vail A, Jovanovic A, Smith CJ (2018). Enzyme replacement therapy and white matter hyperintensity progression in Fabry disease. Neurology.

